# Intussusception of Rectosigmoid Colon Cancer Mimicking a Pedunculated Tumor

**DOI:** 10.1155/2014/696403

**Published:** 2014-05-13

**Authors:** Susumu Saigusa, Masaki Ohi, Hiroki Imaoka, Tadanobu Shimura, Yasuhiro Inoue, Masato Kusunoki

**Affiliations:** ^1^Department of Surgery, Wakaba Hospital, 28-13 Minami-Chuo, Tsu, Mie 514-0832, Japan; ^2^Department of Gastrointestinal and Pediatric Surgery, 2-174 Edobashi, Tsu, Mie 514-8507, Japan

## Abstract

Intussusception in adults is a rare phenomenon involving the colon in approximately 20% of cases. A 65-year-old man was hospitalized with anorexia, anemia, dehydration, and melena. Digital rectal examination revealed a palpable mass approximately 5 cm from the anal verge. The mass moved between the rectosigmoid colon and the rectum below the peritoneal reflection during radiographic examinations and during sigmoidoscopy. We strongly suspected a rectosigmoid pedunculated tumor and performed a low anterior resection. Intraoperatively we observed intussusception of the rectosigmoid colon with easy manual reduction. The tumor was palpable in the rectosigmoid colon. The postoperative course was uneventful. This case illustrates intussusception of a rectosigmoid type 1 colon adenocarcinoma mimicking a pedunculated tumor.

## 1. Introduction

Intussusception is defined as the telescoping of a proximal segment of the gastrointestinal tract into the distal lumen. Intussusception in adults is a rare condition, with colonic intussusception accounting for approximately 20% of the cases [1–3]. Previous reports indicate that computed tomography (CT) is the most sensitive diagnostic modality for intussusception [2, 4]. However, preoperative diagnostic rate is variable [2, 3, 5–7]. Adult intussusception is therefore a challenging diagnostic problem for surgeons.

## 2. Case Report

**Figure 1:**
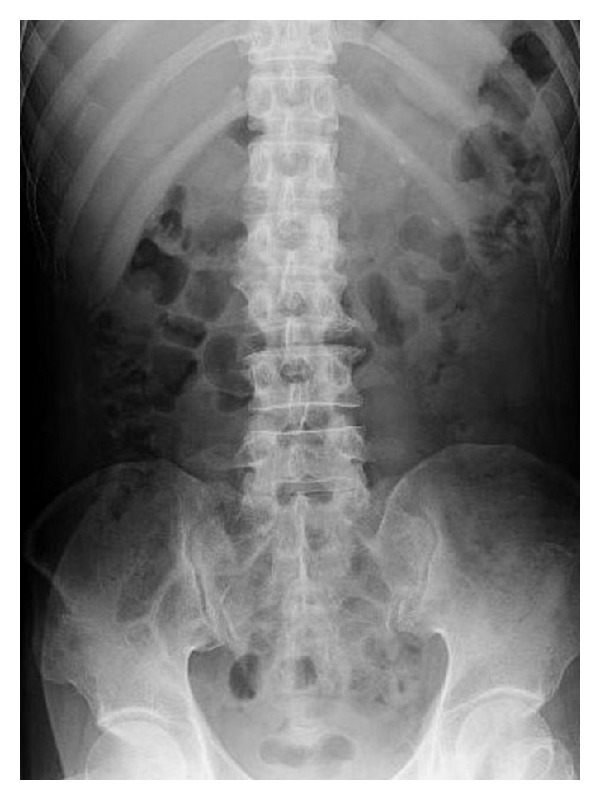
Preoperative abdominal X-ray.

**Figure 2:**
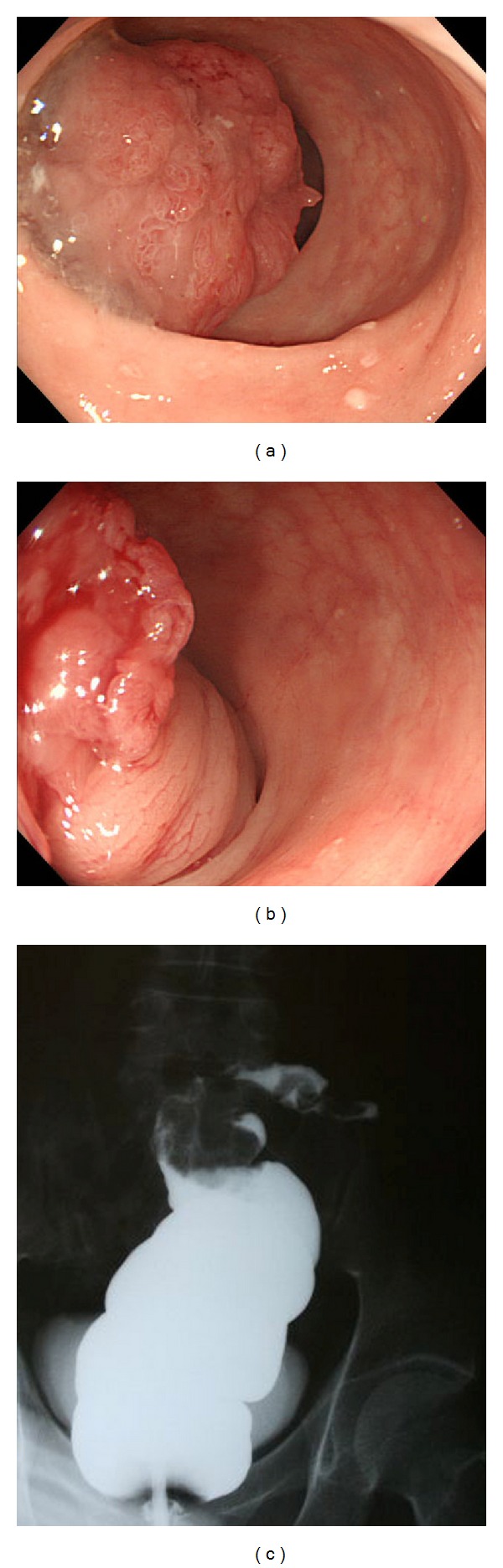
Sigmoidoscopy revealed a tumor occupying the lumen 15 cm from anal verge (a). The bowel mucosa proximal mobile tumor was observed although the camera could not traverse beyond the lesion (b). Gastrografin enema demonstrating the tumor in the rectosigmoid colon without the typical coiled-spring appearance or cup-shape filling defect (c).

**Figure 3:**
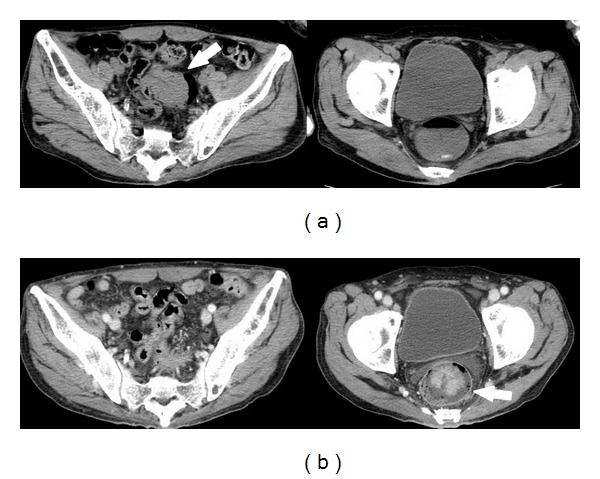
Initial CT showed the tumor located in the rectosigmoid colon (a). Preoperative contrast-enhanced CT showed that the tumor shifted to rectum below the peritoneal reflection (b).

**Figure 4:**
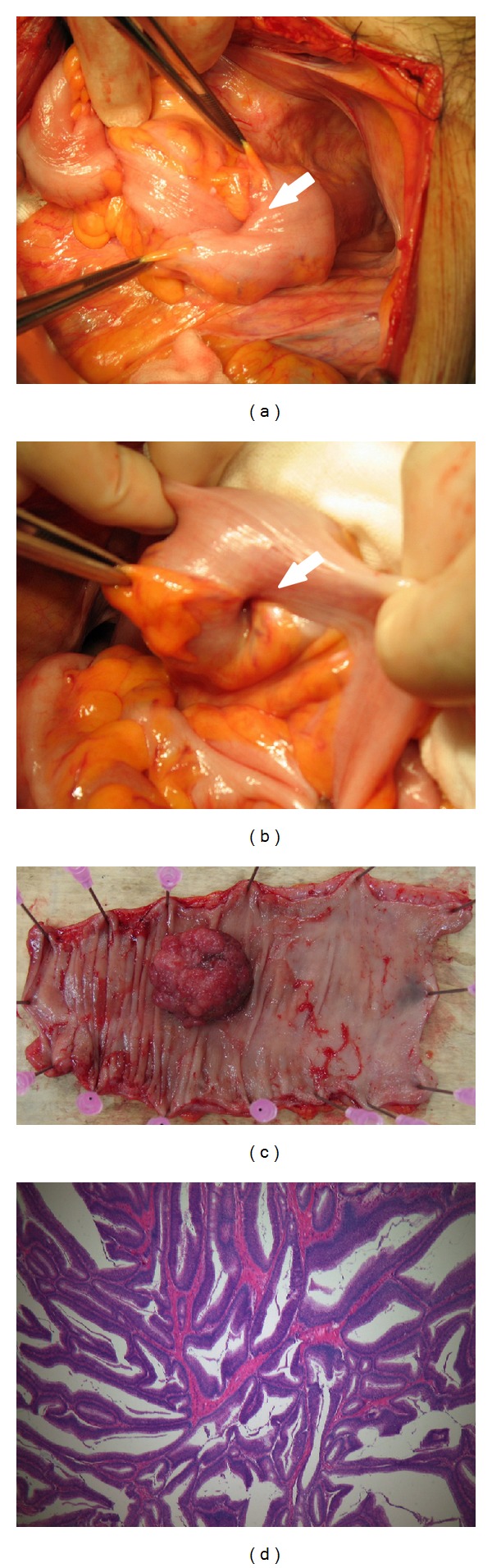
Intussusception of the rectosigmoid colon (a). Suspected area of subserosal invasion (b). Resected specimen with type 1 tumor (35 × 40 mm) (c). Pathological examination revealed a well-differentiated adenocarcinoma with subserosal invasion. Original magnification 40x (d).

A 65-year-old man was hospitalized with appetite loss, anemia, and dehydration. His medical history was significant for alcoholism and gout. Eight days after admission, he had melena without abdominal pain. Laboratory investigation at the time of admission revealed anemia (hemoglobin 9.6 g/dL), hyperuricemia (uric acid 11.0 mg/dL), dehydration (blood urea nitrogen 36 mg/dL, creatinine 1.0 mg/dL, and creatine kinase 1790 IU/l), mild inflammation (C-reactive protein 3.41 mg/dL), and elevated liver enzymes (aspirate aminotransferase 153 IU/l, alanine aminotransferase 119 IU/l). Abdominal X-ray was unremarkable (Figure 1). After the occurrence of melena, digital rectal examination revealed a palpable mass approximately 5 cm from the anal verge. Sigmoidoscopy revealed a mobile tumor occupying the lumen 15 cm from anal verge, the proximal side of which could not be visualized (Figures 2(a) and 2(b)). Biopsy revealed well-differentiated adenocarcinoma. Gastrografin enema and initial CT showed that the tumor was in the rectosigmoid colon (Figures 2(c) and 3(a)). Subsequent contrast-enhanced CT showed that the tumor shifted into the rectum below the peritoneal reflection with no evidence of distant metastasis (Figure 3(b)). The patient did not have any obstructive symptoms. We strongly suspected a rectosigmoid pedunculated tumor. After informed consent, definitive surgery was performed. Operative findings confirmed intussusception of the rectosigmoid colon with easy manual reduction (Figure 4(a)). The tumor was palpable in the rectosigmoid colon (Figure 4(b)). A low anterior resection with a stapled anastomosis was performed. Histopathological examination revealed a 4 cm, T3N0 M0 (stage II), well-differentiated adenocarcinoma (Figures 4(c) and 4(d)). The patient's postoperative course was uneventful.

## 3. Discussion

The rate of preoperative diagnosis of intussusception has improved with advancement of imaging studies but continues to be challenging [2, 3, 5–7]. Previous reports indicate that CT is the most sensitive diagnostic modality for intussusception [2, 4]. However, preoperative CT did not reveal the typical appearance of intussusception such as a pseudokidney or target sign in our case. The intussusception in our case had spontaneously reduced, and our intraoperative observation confirmed that it was easily repositioned by traction of the proximal colon.

Several authors have reported laparoscopic management of sigmoidorectal intussusception [8–10]. Greenley et al. indicated that an extra skill set is needed to reduce the intussuscepted segment of colon without violating the lumen of the bowel in sigmoidorectal intussusception and that a combined laparoscopic and perineal attempt at reduction followed by resection is justified if there is no evidence of bowel necrosis, inflammation, or tumor invasion [10]. En bloc resection without reduction is recommended for colonic intussusception [11–13] as colonic intussusception is more likely to have a malignant etiology [2, 14, 15] and bowel injury including perforation in malignant cases has a high risk of cancer cell dissemination [1]. We performed laparotomy but not laparoscopic approach in the present case because we suspected a pedunculated tumor moving into the rectum and wanted to confirm the tumor location by direct palpation.

Macroscopic and pathological features of intussusception caused by colon adenocarcinoma of the colon have not been well evaluated. Several reports have shown that the lead points of colonic intussusception were mostly due to villous or type 1 (protruded type; Bormann's classification type 1) tumors [10, 16–20]. Chen et al. reported synchronous double colocolic intussusception and that both adenocarcinomas revealed macroscopic type 1 or villous adenocarcinomas with subserosal invasion [21]. In the present case report, we also found that type 1 adenocarcinoma with subserosal invasion resulted in colonic intussusception. Warshauer and Lee indicated that neoplastic lead points were significantly longer and larger in diameter than nonneoplastic ones [22]. Taken together, colonic intussusception may be due to large macroscopic type 1 or villous tumors.

In conclusion, intussusception of rectosigmoid colon adenocarcinoma can mimic a pedunculated tumor. Preoperative diagnosis of intussusception with spontaneous reduction is challenging.
